# Supplementary figures and images for: CTP sensing and Mec1ATR-Rad53CHK1/CHK2 mediate a two-layered response to inhibition of glutamine metabolism
Source: PLoS Genet. 2022 Mar 3;18(3):e1010101. doi: 10.1371/journal.pgen.1010101 (PMC8923462; doi:10.1371/journal.pgen.1010101)

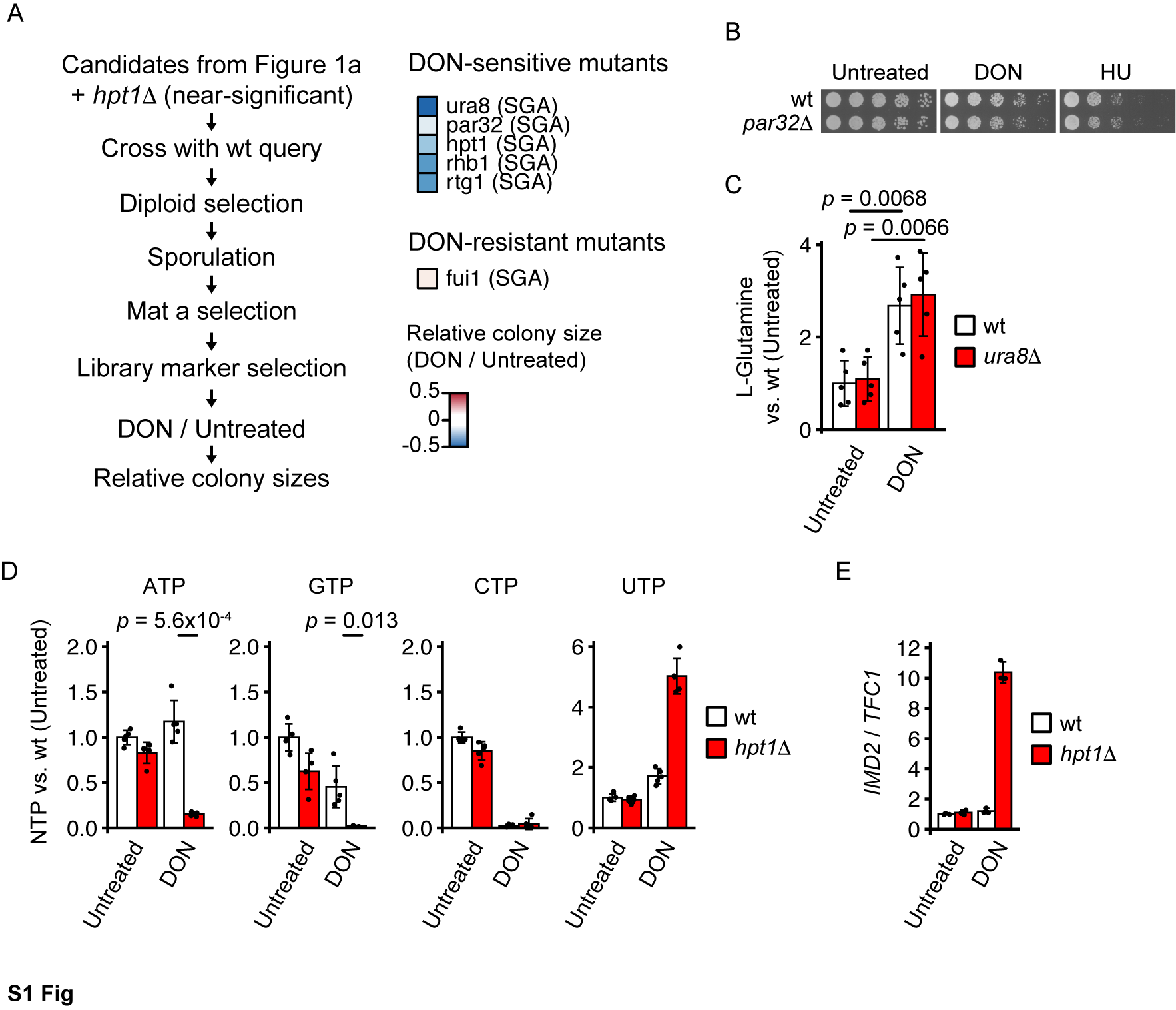

Supplement: S1 Fig — (A) Schematic of the validation screen for DON resistance genes. The heatmaps represent the difference between DON-treated vs. untreated colony size. All values are log2-transformed, corrected for standard deviation and normalized to the 70-percentile of control clones. Statistical information is provided in S1 Table. (B) 107 cells / mL of the indicated genotype in the S288C background were serially diluted (1:6), spotted on YPD plates with or without 250 μM DON or 200 mM HU and grown for 2d (Untreated), 3d (DON) or 4d (HU). (C, D) Exponentially growing cells of the indicated genotypes in the W303 (C) or S288c (D) background were treated with 300 μM DON for 2h as indicated. Metabolites were extracted and glutamine levels were quantified by nano-LC-MS/MS. Values are ion intensities (peak areas) normalized by the mean of untreated wt samples. Significance was calculated by Student’s t-test (two-sided) over 5 replicate cultures. (E) Exponentially growing cells of the indicated genotypes in the S288c background were cultured in YPD and treated with 300 μM DON for 2h. Samples were collected to quantify IMD2 mRNAs by RT-qPCR using TFC1 as reference (n = 3 independent replicate cultures). Representative spot assay images are shown. wt = wildtype. (TIF) [file pgen.1010101.s001.tif]

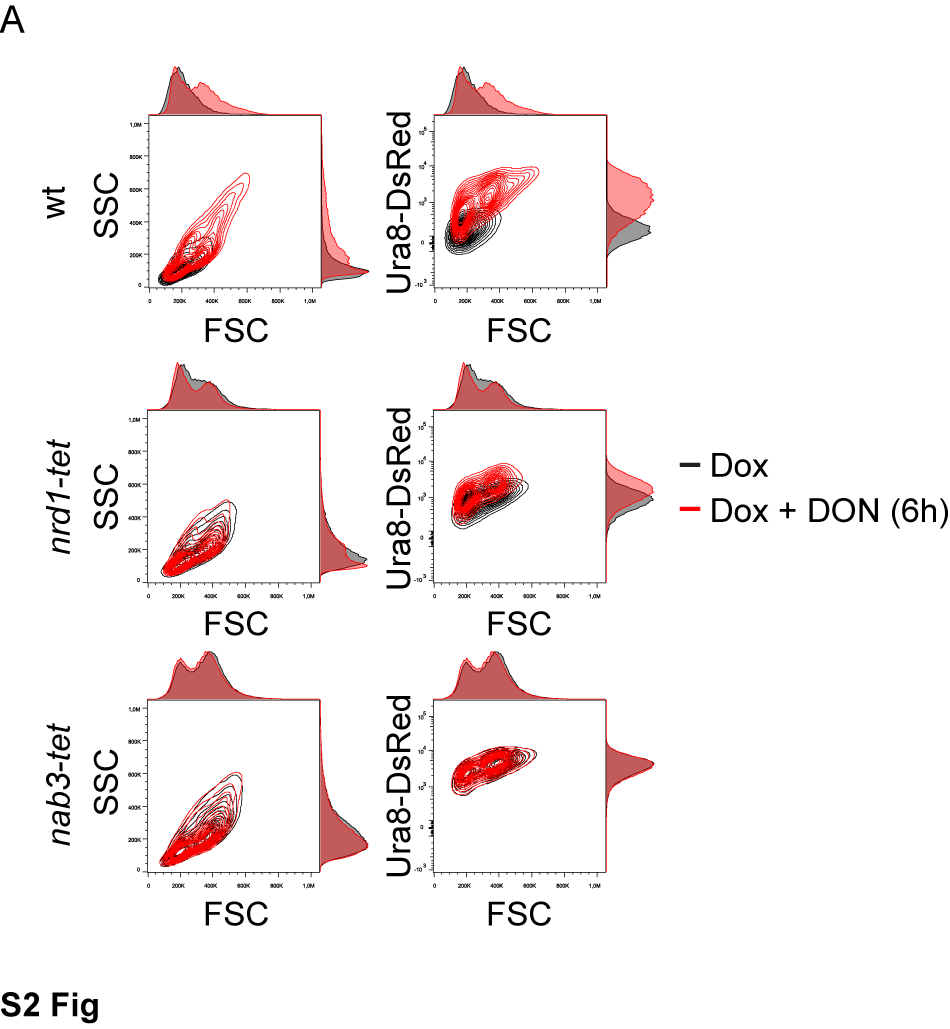

Supplement: S2 Fig — (A) Strains with doxycycline-repressible alleles of the Nrd1 complex (nrd1-tet, nab3-tet) from the Tet-Promoters Hughes library were crossed with a query strain expressing endogenous DsRed-tagged Ura8 and GFP-tagged Rnr3. Mutant offspring was cultured overnight in YPD with 50 μM doxycycline, treated with 300 μM DON in YPD with 50 μM doxycycline and fixed with formaldehyde after 6h. Expression of the tagged proteins was analyzed by flow cytometry (n = 3 independent replicate cultures). The plots are from the samples in Fig 5G and provide additional information on cell parameters. (TIF) [file pgen.1010101.s002.tif]

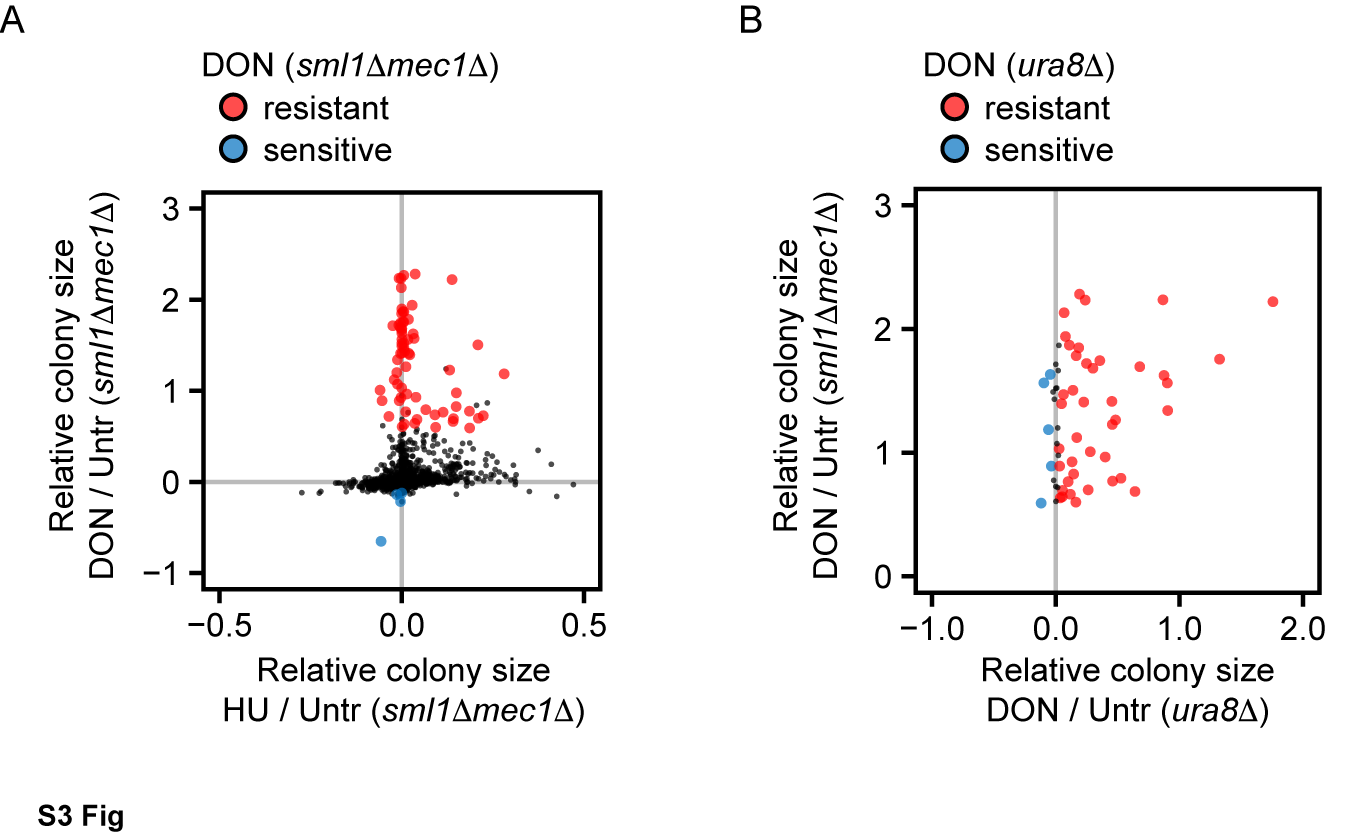

Supplement: S3 Fig — (A, B) The viable haploid gene deletion yeast library was crossed with sml1Δ, sml1Δmec1Δ, wt and ura8Δ query strains. Mutant offspring was selected, replicated on YPD (Untreated), YPD + 200 μM DON or YPD + 150 mM HU, and colony sizes were quantified. Each dot represents a library mutant. The x and y axes in (A) represent the relative colony sizes in the sml1Δmec1Δ background on YPD + HU vs. YPD (x) or YPD + DON vs. YPD (y) after 70-percentile normalization to all other sml1Δmec1Δ colonies with the same treatment, respectively. In (B) only the DON sensitivity suppressors from (A) are shown. The x and y axes in (B) represent the relative colony sizes in the ura8Δ (x) and sml1Δmec1Δ (y) backgrounds on YPD + DON vs. YPD after 70-percentile normalization to all other ura8Δ and sml1Δmec1Δ colonies, respectively. All values are log2-transformed and adjusted for standard deviation. Significant sensitivity or resistance to DON in the sml1Δmec1Δ (A) or ura8Δ (B) background (see Materials and Methods) is indicated in blue (sensitive) and red (resistant). Statistical information is provided in S3 and S4 Tables. (TIF) [file pgen.1010101.s003.tif]
