# Supplementary material for: CTP sensing and Mec1ATR-Rad53CHK1/CHK2 mediate a two-layered response to inhibition of glutamine metabolism
Source: PLoS Genet. 2022 Mar 3;18(3):e1010101. doi: 10.1371/journal.pgen.1010101 (PMC8923462; doi:10.1371/journal.pgen.1010101)
Supplement: Attachment — Submitted filename: Response to Reviewers - Ajazi et al., 2022.docx [file pgen.1010101.s010.docx]

**Detailed response to the guest editor**

Answers are provided in red

Sections cited from the new manuscript are with yellow background

The guest editor felt that it would be important to show that deleting HPT1 confers a strong reduction in AMP/GMP levels and/or a strong induction of IMD2 transcription in cells treated with DON, to confirm the presumed mechanism of DON-sensitivity on deletion of HPT1.

To clarify this point we measured the NTP levels in wt and *hpt1Δ* mutants before and after the glutamine analog (DON) treatment for 2 hours. We found that *HPT1* deletion strongly reduced ATP and GTP levels during DON treatment compared to wt cells (Supplementary Figure 1D in the revised version of the manuscript). We further measured the expression levels of the GTP-repressible *IMD2* gene by RT-qPCR and found that DON treatment strongly induced *IMD2* in *hpt1Δ* mutants (Supplementary Figure 1E in the revised version of the manuscript). Together, this supports the idea that Hpt1 is required during DON exposure to maintain purine NTP levels. We thank the guest editor for suggesting us to clarify the mechanism by which *HPT1* mediates resistance to DON.

He/she also felt that categorization of the suppressors of DON toxicity based on their effects on UTP8 and RNR3 reporter expression +/- DON was not as clear-cut as suggested by the authors, and certain suppressors that don’t fall into either group were not discussed at all, calling for a more conscientious description of these data.

We agree that we over-simplified the conclusions of our flow cytometry screen. We modified the conclusion paragraph to raise awareness that such clear-cut classification of suppressors does not provide a full explanation:

In summary, our data suggest that DON hypersensitivity suppressors can act through at least two mechanisms: shifting the intracellular DON-to-glutamine ratio and reducing DNA break formation. However, we note that the suppression mechanisms are more complex than depicted here. First, not all amino acid transport regulators clearly suppress Ura8 induction (Fig 6C), which could imply additional functions of the candidates independent from CTP regulation. Second, we also identified several candidates belonging to diverse biological processes such as translation, histone deacetylation and lipid metabolism. The role of these candidates in the DON response requires further investigation to better understand the full spectrum of suppression mechanisms.

Thirdly, it was felt that the analysis of these suppressors was quite preliminary and that a few follow-up experiments were required to show that representative suppressors of the first group actually do boost CTP levels in the presence of DON, whereas representative suppressors of the second group reduce the formation of ds DNA breaks (if this is indeed the presumed mechanism, which was not explicitly stated but should have been.)

To address this point, we chose representative candidates of the two groups. For the first group of suppressors with low Ura8 and Rnr3 induction after DON treatment mediating DON resistance potentially by boosting CTP levels we used *gnp1Δ* mutants (representative of amino acid transporters) and *gtr1Δ* mutants (representative of TORC1 activators). We validated the suppression of DON sensitivity by these mutations (Figure 6D in the revised version of the manuscript) and measured the CTP levels by LC-MS/MS. We found that deletion of *GNP1* and *GTR1* increased the intracellular CTP levels under DON treatment (Figure 6E in the revised version of the manuscript), further suggesting an involvement of the glutamine transporter Gnp1 and TORC1 activity in targeting of CTP metabolism by DON.

As correctly stated by the guest Editor, the second group of suppressors that show similar Ura8 to wt cells but low Rnr3 induction after DON treatment likely confers DON resistance by preventing DNA breaks. We selected the representative genes *CDC73* (PAF complex), *YKE2* (prefoldin complex), *ELP6* (elongator complex) and *RTR1* (RNA polymerase II phosphatase) and validated suppression of DON sensitivity in the deletion mutations (Figure 6F in the revised version of the manuscript). Deletion of all four candidate genes further suppressed DON-induced chromosome breaks in *sml1Δmec1Δ* mutants as measured by PFGE (Figure 6G in the revised version of the manuscript). We included a new paragraph in the Discussion section on the suppression of DNA breaks by deletion of the tested candidates (Involvement of transcription in DON-induced DNA breakage).

These new data strengthen our hypothesis that DON hypersensitivity suppressors can act mainly through two distinct mechanisms: shifting the intracellular DON-to-glutamine ratio hence boosting CTP levels and preventing DNA break formation.

We thank the guest editor for suggesting us to perform these additional experiments, whose results contributed to better clarify the role of these candidates in mediating DON sensitivity.

**Detailed response to the Reviewers**

Answers are provided in red

Sections cited from the new manuscript are with yellow background

Reviewer #1: The manuscript entitled « CTP sensing and Mec1ATR-Rad53CHK1/CHK2 mediate a two-layered response to inhibition of glutamine metabolism” by Ajazi and co-workers reports a chemogenetic analysis of the toxicity of a glutamine analog 6-diazo-5-oxo-L-norleucine (DON) in Saccharomyces cerevisiae. The authors identified several genes affecting DON resistance when mutated, they performed epistasis studies and focused on one of them URA8 which encodes an isoform of CTP synthase. They show that the other isoform (URA7) is less important for DON resistance although it is the major isoform. They establish that URA8, although contributing the minority isoform under standard growth conditions, is strongly induced under CTP deprivation, as for example when cells are treated with DON, and thereby is critical for resistance to the glutamine analog. The authors decipher a CUT-dependent mechanism of transcriptional ‘antitermination’ based on a repetition of Cs in the URA8 upstream region and the Nrd1/Nab3 complex. Finally, they show that in a ura8 background, DON treatment is associated to a DNA damage response and they screened for suppressors of this phenotype and this way identified mutants in the TOR pathway. This work gives an overview on the glutamine deprivation response and its connection to pyrimidine metabolism and DNA damage. This is a nice and solid piece of work. The experiments are sound, the results are clear and support the conclusions.

We would like to thank the Reviewer for the positive comments about our work.

Minor point:
The experiments were done on YPD containing uracil and adenine. It would be interesting to compare the effect of DON on the mutants (most specifically ura8 and hpt1) on media containing or not uracil and adenine.

We agree we the Reviewer that it would be interesting to see the effect of DON in the absence of uracil and adenine. However the yeast backgrounds used for all mutants in this study (S288c, W303) are both defective for the *de novo* synthesis of UMP, carrying a mutation in *URA3* gene. Therefore, the growth in a synthetic medium containing no uracil is impossible. The same applies to the purine *de novo* synthesis pathway in our W303 background, in which most experiments with the *ura8Δ* mutation have been performed, because this background carries a mutation in the *ADE2* gene, rendering exogenously supplied adenine essential. In the revised version of the manuscript, we have now performed NTP quantification in both backgrounds, and we consistently observe that DON also reduces GTP levels in the S288c background but not (or only mildly) in the W303 background (compare W303 in Figure 1I with S288c in the new Supplementary Figure 1D). We believe that this is due to the adaptation of the W303 background to the usage of the purine salvage pathway, whereas the S288c background can also perform de novo purine synthesis. We agree that our results need to be interpreted in the context of an active salvage pathway.

Reviewer #2: The manuscript from Ajazi et al. describes genetic interactions with the glutamine analog DON in budding yeast. Their results are of practical interest since pro-drug derivatives of DON are currently being tested as cancer therapies in animal studies, and the physiologically relevant molecular targets of DON are diverse. The genetic screens employed were limited to deletions of non-essential genes, but nevertheless yielded interesting results. A focus of the study is the surprising finding that deletion of one of two CTP synthetase genes, URA8, confers sensitivity to DON while deletion of the other, URA7, does not. (Glutamine is the source of nitrogen for the conversion of UTP to CTP by CTP synthetase.) In investigating the basis of this difference, the authors found that Ura8 protein is induced by glutamine or CTP depletion but Ura7 is not, and that induction occurs at the level of Ura8 mRNA synthesis. They therefore conclude that Ura8 confers resistance to DON via its increased expression, overcoming covalent inactivation of the enzyme by DON.

We thank the Reviewer for his positive comments on our manuscript.

This mechanism is similar to the resistance to mycophenolic acid (MPA) conferred by Imd2, but not by Imd3 and Imd4, except that Imd2 is also intrinsically resistant to MPA in addition to being induced. The authors do not investigate, or even consider, if Ura8 is intrinsically resistant to inactivation by DON. I am skeptical that induction of Ura8 to a level only slightly higher than constitutive Ura7 expression would confer DON resistance by mass action alone. The author should acknowledge this issue and, if possible, address it experimentally.

We thank the Reviewer for this constructive suggestion. We addressed the point by citing the relevant study and performing an experiment to assess differences in the capacity of Ura7 and Ura8 to confer DON resistance. We added the following section to the manuscript:

An intrinsic resistance of the Ura8 enzyme towards DON inhibition could contribute to its specific requirement for survival during DON exposure; a similar mechanism has been previously described for the IMP dehydrogenase Imd2 which confers resistance to the IMP dehydrogenase inhibitor mycophenolic acid, through a combination of its gene inducibility and lower enzyme inhibition in comparison to other IMP dehydrogenase isoforms [34]. We therefore tested if Ura7 and Ura8 had different capacities to restore DON resistance when over-expressed at comparable levels in *ura8Δ* cells. We achieved similar expression levels of Ura7 and Ura8 (Fig 1J), and we found that both CTP synthases similarly restored DON resistance to wt levels (Fig 1K). This observation suggests that likely Ura8 inducibility rather than an intrinsic property of Ura8 accounts for its role in DON resistance, although a characterization of Ura8 and Ura7 enzymatic activities during DON treatment would be required to fully assess inherent inhibition resistance.

We further added the following sentence to the Discussion:

Differences in enzyme inactivation by DON or different glutamine Km values could further contribute to the different requirement of Ura8 and Ura7 for resistance towards inhibition of glutamine metabolism, and additional biochemical experiment would be required to further investigate these possibilities.

The authors unfamiliarity with the IMD2 and Nrd1-Nab3-Sen1 (NNS) termination literature causes them to misinterpret their results in other ways as well.

We apologize for not considering the IMD2 literature in our manuscript. This has led to incomplete integration of our work in the literature context. We have addressed this issue by citing the relevant literature, changing the line of argumentation in Results and Discussion and modifying the model. This has led to significant improvements of the manuscript. Please see specific comments below.

In the Discussion (pg 17, line 4) the authors state: “So far it was not known if limitation of any NTP triggers a global induction for all CUT-regulated nucleotide synthesis enzymes, or if each NTP could regulate its respective synthesis pathway specifically.” This statement is incorrect.

We now introduce this discussion point as follows:

The GTP sensing mechanism at the *IMD2* locus suggests that NTP shortage responses are highly NTP-specific and are not part of a general NTP response: GTP shortage specifically induces *IMD2* expression and therefore enhances GTP synthesis [43].

We then describe that our study provides insight into CTP sensing, and how far URA8 induction during DON treatment is comparable to the GTP sensing at the IMD2 locus.

The authors need to familiarize themselves with the literature on yeast IMD2 regulation from the Brow and Reines labs published in 2008, which indicates that IMD2 is regulated by alternative start site selection that is dependent on intracellular GTP concentration.

We have included several relevant studies from both laboratories in our manuscript. We agree that this was important to establish the correct line of argumentation, and we believe that the updated version of the manuscript integrates much better with the existing literature.

The upstream CUT start sites are at GG dinucleotides or GGG trinucleotides and are recognized when intracellular GTP is replete. The downstream mRNA start site is an AA dinucleotide. Thiebaut et al. 2008 (ref. 42, Fig. 6) mapped the URA8 upstream CUT starts to six sites, five of which are C nucleotides and the sixth is a T followed by a C, while the URA8 mRNA start sites are purines. Pyrimidine start sites are unusual for Pol II, so this pattern is striking and suggests that URA8 may be regulated by the same mechanism as IMD2 but sensing CTP rather than GTP concentration. The oligoC stretch mutated by the authors (Figure 5D) encompasses two of the start sites identified by Thiebaut et al. 2008 and the loss of induction observed supports an IMD2-like mechanism that is different from the mechanism proposed by the authors (Figure 5E).

The suggestion that URA8 regulation may also be mediated by NTP-sensitive start site selection is very interesting, and we built our argumentation in the Results section around this mechanism. The start site selection model is consistent with most of our data, and also with the data presented in Thiebaut et al., 2008. In the new manuscript we consider start site selection as a mechanism mediating the CTP sensing. We changed the working model (Figure 5E) accordingly. However, Thiebaut et al., 2008 also show that CUT levels are not consistently reduced under conditions which induce expression of *URA2* or *URA8*, which would be expected from a pure start site selection mechanism. This led the authors of Thiebaut et al., 2008 to the proposal of two alternative highly complex models (Figure 7) that consider a potential role of the R box in promoting main ORF transcription during NTP limitation. Although we agree with this Reviewer that the start site selection mechanism is generally more elegant and simplistic, we also see that the above data cannot be fully explained by this mechanism. In our own experiments (Figure 5F) we observe that C-to-G mutation in the CUT on the one hand spontaneously induces *URA8* expression, which is consistent with the start site selection mechanism. On the other hand, the same mutation also prevents the full de-repression of *URA8*, which supports a more complex model for *URA8* regulation as suggested by Thiebaut et al. 2008. We have integrated both considerations in the manuscript (all relevant text modifications are marked in yellow), and we hope that this Reviewer agrees with us that the new line of argumentation is more adequate considering our data and the literature.

The authors’ proposed mechanism does not make sense to me. How would “crowding” of transcribing Pol II on the DNA displace the NNS complex from the nascent transcripts? It is scanning Pol II, not transcribing Pol II, that is responsive to GTP concentration on the IMD2 gene and, likely, on the URA8 gene.

We agree with the Reviewer and we removed the crowding from the working model (Figure 5E). We thank the Reviewer for this suggestion and we believe that the new working model is less speculative and in agreement with the published literature.

Furthermore, the effect of NNS mutants on URA8 mRNA synthesis has already been examined by the Brow lab (Chen et al. 2017, Fig. S5). This study revealed the stronger effect of decreased Nab3 function compared to that of Nrd1, as is also seen by the authors (Fig. 5G). In general, the manuscript should be rewritten to better connect the current results with the published data for both IMD2 and URA8.

We now cite the above study investigating the termination defects of NNS mutants:

These observations are in agreement with a previous study showing that CUT transcription termination defects at the *URA8* CUT under condition of abundant NTPs are more severe in *nab3* mutants than in *nrd1* mutants [45].

We further use the study as a reference when introducing the role of Nrd1/Nab3 in CUT termination in the context of NTP sensing:

These CUTs are produced when NTPs are abundant, and their production involves transcription termination through the Nrd1/Nab3 complex which prevents progression of RNA polymerase II into the main ORF [41-45].

The experiments on effects of DON on the DDR pathway generally add value to the manuscript, although I question the identification of Rtg1 as a homolog (or even analog) of Myc.
We agree with this Reviewer that the link between Rtg1 and Myc is not as clearly supported by the literature as it may appear from the use of the Rtg1^Myc^ nomenclature. Since the link between Rtg1 and Myc is not important for the conclusions of the manuscript, we removed all comparisons of Rtg1 and Myc throughout the manuscript.

Additional comments:

1) Figure 1H and elsewhere: arrows meant to indicate decreased levels should be placed alongside the compound (here, CTP) rather than above or below it, to prevent confusion with a metabolic pathway.

We thank the Reviewer for the suggestion, and we adjusted the Figures accordingly.

2) Page 7, lines 9-12: The difference in sensitivity of ura7-delta and ura8-delta to MSX (Fig. 2B) is considerably less than for DON (Fig. 1E) and may simply suggest that Ura8 has a lower Km for glutamine.

We agree that the sensitivity data alone could be explained by a lower Km of Ura8 for glutamine. However, the main focus of Figure 2 is the induction of URA8 expression by MSX (in analogy to DON); the sensitivity data are then provided because hyper-sensitivity of ura8-delta mutants are a prediction of the induction data presented in Figure 2A. To raise awareness of this alternative interpretation, we added the following sentence to the discussion:

Differences in enzyme inactivation by DON or different glutamine Km values could further contribute to the different requirement of Ura8 and Ura7 for resistance towards inhibition of glutamine metabolism, and additional biochemical experiment would be required to further investigate these possibilities.

3) Figure 2B: the “sml1-delta” notation in this panel seems spurious. If not, it should be explained.

We agree with the Reviewer, and we changed the notation to the commonly used notation.

4) The Figure 3A legend should read “with or without 300 uM DON”.

We agree with the Reviewer, and we changed the Figure legend accordingly.

5) Page 12, lines 11 and 12: IMD2 mRNA synthesis is regulated by GTP, not UTP (see above).

We thank the Reviewer for all of the comments related to GTP sensing. We corrected this statement.

6) Page 16, line 42: the NNS termination complex does not “interrupt the synthesis of an upstream CUT”. It creates a CUT in place of an mRNA.

We agree that this expression is not adequate and corrected this type of statement throughout the manuscript. We thank the Reviewer for pointing out this issue.

We would like to thank this reviewer for his extensive constructive criticism which led to the significant improvement of the manuscript!

Reviewer #3: CTP sensing… Ajazi et al NAR

This is a thorough study examining the toxicity of glutamine analog called DON. The general motivation for this study is the examination of why glutamate analogs like DON are so toxic to cells. DON is a useful cancer therapeutic tool. The authors take advantage of the genetic and molecular tools in budding yeast, which is also sensitive to DON. Glutamine analogs (e.g. DON), which compete with glutamine as competitive inhibitors are toxic to cells due in part to inhibition of CTP synthase (encoded by two genes, URA7 and URA8). There are many cool details of the regulation of URA8 and URA7 that keeps the reader on his/her/their toes. The first screen searches for increased sensitization to DON treatment, and five genes are identified and studied. The second screen, introduced and analyzed in full in the last figure, searches for DON resistance or sensitivity in a mec1 sml1 mutant (defective in DDR responses). The model in Figure 7 reassures the reader that they got the story and the model straight, incorporating some of the genes identified into a conceptually coherent model.
This study has a lot in it, and specific studies seem well done and convincing. I reviewed this paper for another journal, I made several suggestions which the authors seemed to have followed. Aspects of the study remain a worthy challenge to follow conceptually, because there is a lot here. The writing seems clearer in this version, for which the readers will be thankful. And the Discussion is clearer to more readers than the earlier version. The data are spectacular. This paper is likely to provide a plethora of insights into how metabolic studies can be done; the full implementation of genetic techniques possible in yeast are on full display.

We thank the Reviewer for his very positive comments on our manuscript.

Minor Comments.
1. Fig 1J is …interesting. Sort of like it.

We would like to thank the Reviewer for the positive comment.

2. Fig 5E. Love the model.

We would like to thank the Reviewer for the positive comment.

Yet, if they are showing the DNA and what is being transcribed, should not the oligoC be changed to oligoG as the template RNA polymerase is reading and stalling when CTP is low?

We now added the notion “The coding strand DNA sequence is represented.” in the Figure legend. This clarifies that we do not display the DNA strand that the RNA polymerase II copies from, but the DNA strand that has the same sequence as the synthesized mRNA. This clarification may avoid confusion and we thank the Reviewer for pointing out this potential issue.

**Have all data underlying the figures and results presented in the manuscript been provided?**
Large-scale datasets should be made available via a public repository as described in the *PLOS Genetics* [data availability policy](http://journals.plos.org/plosgenetics/s/data-availability), and numerical data that underlies graphs or summary statistics should be provided in spreadsheet form as supporting information.

Reviewer #1: Yes

Reviewer #2: Yes

Reviewer #3: Yes

PLOS authors have the option to publish the peer review history of their article ([what does this mean?](https://journals.plos.org/plosgenetics/s/editorial-and-peer-review-process#loc-peer-review-history)). If published, this will include your full peer review and any attached files.


If you choose “no”, your identity will remain anonymous but your review may still be made public.


**Do you want your identity to be public for this peer review?** For information about this choice, including consent withdrawal, please see our [Privacy Policy](https://www.plos.org/privacy-policy).

Reviewer #1: No

Reviewer #2: No

Reviewer #3: No
